# Supplementary material for: MicroRNA-4516 in Urinary Exosomes as a Biomarker of Premature Ovarian Insufficiency
Source: Cells. 2022 Sep 7;11(18):2797. doi: 10.3390/cells11182797 (PMC9497098; doi:10.3390/cells11182797)
Supplement: Supplementary file 1 [file cells-11-02797-s001.zip › cells-1863144-supplementary.pdf]

cells MDPI

Supplementary Material

Table S1. Selected 12 microRNAs from two groups

Group1	*miRNAs that were highly differentially expressed in the POI or Turner syndrome group*
	hsa-miR-16-5p, hsa-miR-29a-3p, hsa-miR-30b-5p, hsa-miR-151a-5p, hsa-miR-423-3p, hsa-miR-4516
Group2	*substantial differences in expression in the POI or Turner syndrome group, but evenly expressed within the same patient group (e.g., the POI group or Turner syndrome group)*
	hsa-miR-20a-5p, hsa-miR-99b-3p, hsa-miR-200b-3p, hsa-miR-941, hsa-miR-4492, hsa-miR-7847-3p

Table S2. Identification of miR-4516 targets using miRTarBase2020

ID	Target	Validation methods								Sum	paper
		Strong evidence			Less strong evidence						
		Reporter assay	Western blot	qRT-PCR	Microarray	NGS	qSILAC	Others	CLIP-Seq		
MIRT054631	STAT3	V	V	V	V					4	1
MIRT100578	PIM1					V				1	1
MIRT1309664	BRD3					V				1	0
MIRT1309917	PSMD5					V				1	0
MIRT1310131	ARSD					V				1	0

Abbreviations (NGS: next-generation sequencing, qSILAC: quantitative Stable isotope labeling by amino acids in cell culture)

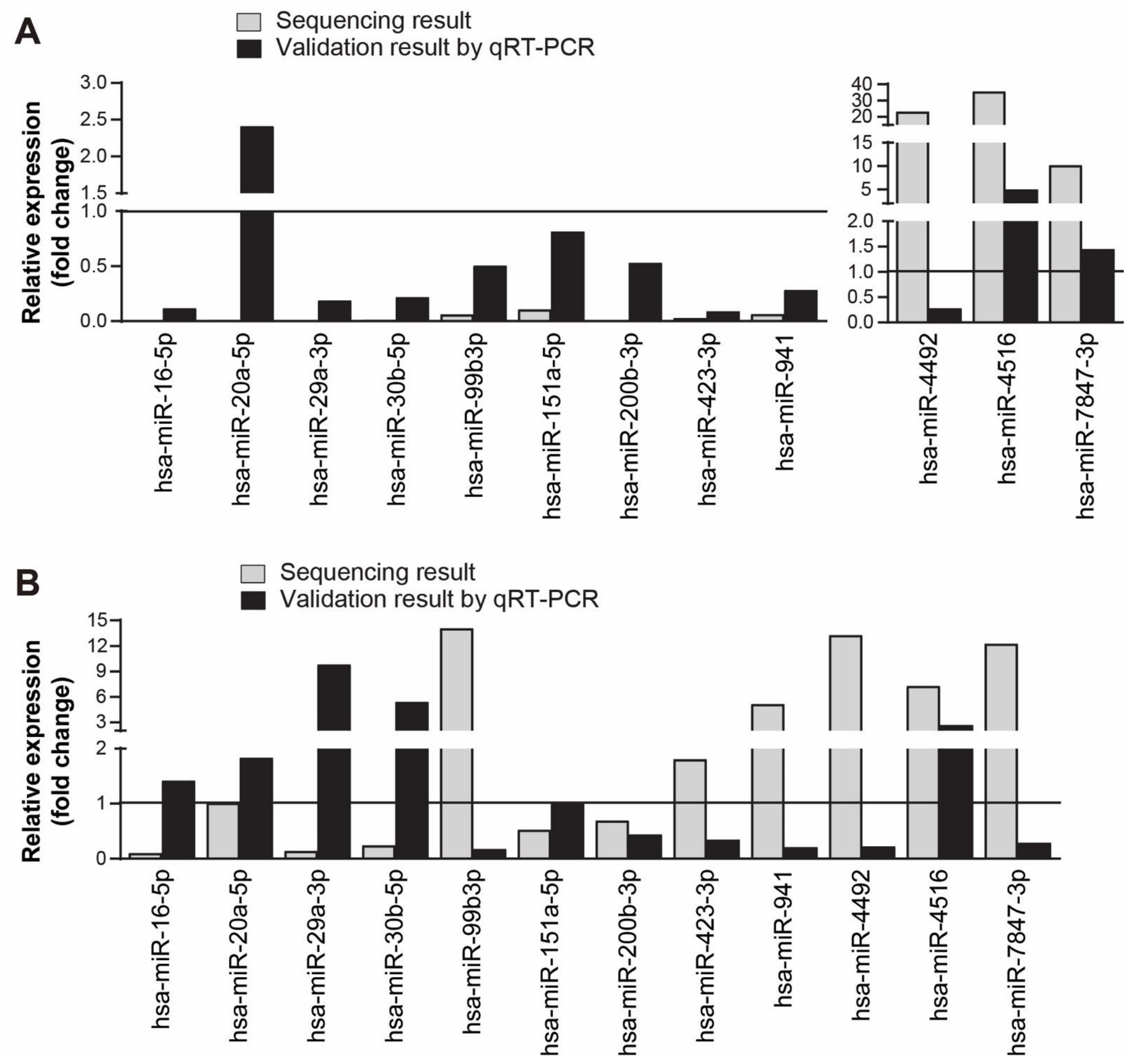

Figure S1. Comparison of the relative expression patterns of selected microRNAs obtained from the mRNA sequencing and qRT-PCR validation. (A) Relative expression levels of 12 differentially expressed miRNAs were identified in patients with premature ovarian insufficiency (POI) using the mRNA sequencing (POI, n = 7; control, n = 5) and validated using the qRT-PCR (POI, n = 15; control, n = 20). (B) Relative expression levels of 12 differentially expressed miRNAs were identified in patients with Turner syndrome using the mRNA sequencing (Turner, n = 7; control, n = 5) and validated using the qRT-PCR (Turner, n = 11; control, n = 20). Relative miRNA expression determined using qRT-PCR was normalized with UniSp6 as a control. Asterisks (*) indicate significant differences (P<0.05) compared with the control group.

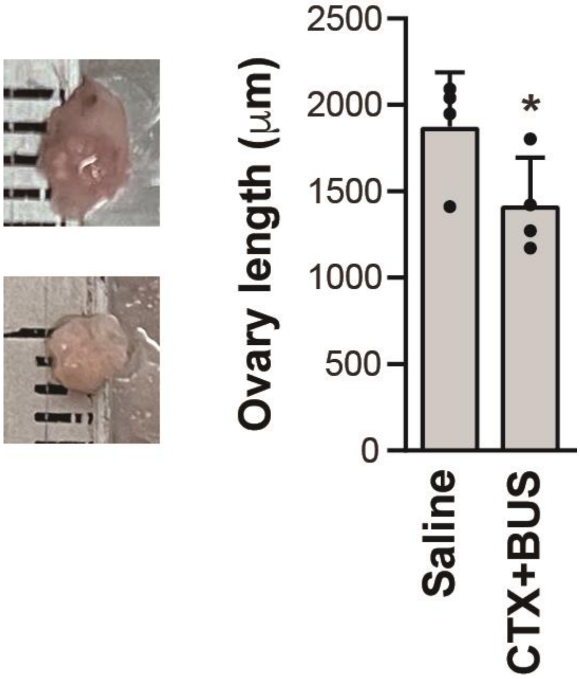

Figure S2. Representative images of ovaries from saline-injected control (Saline) and cyclophosphamide (CTX) + busulfan (BUS)-injected POI mice (CTX+BUS). The ovary length was quantified using ImageJ software (n = 4).

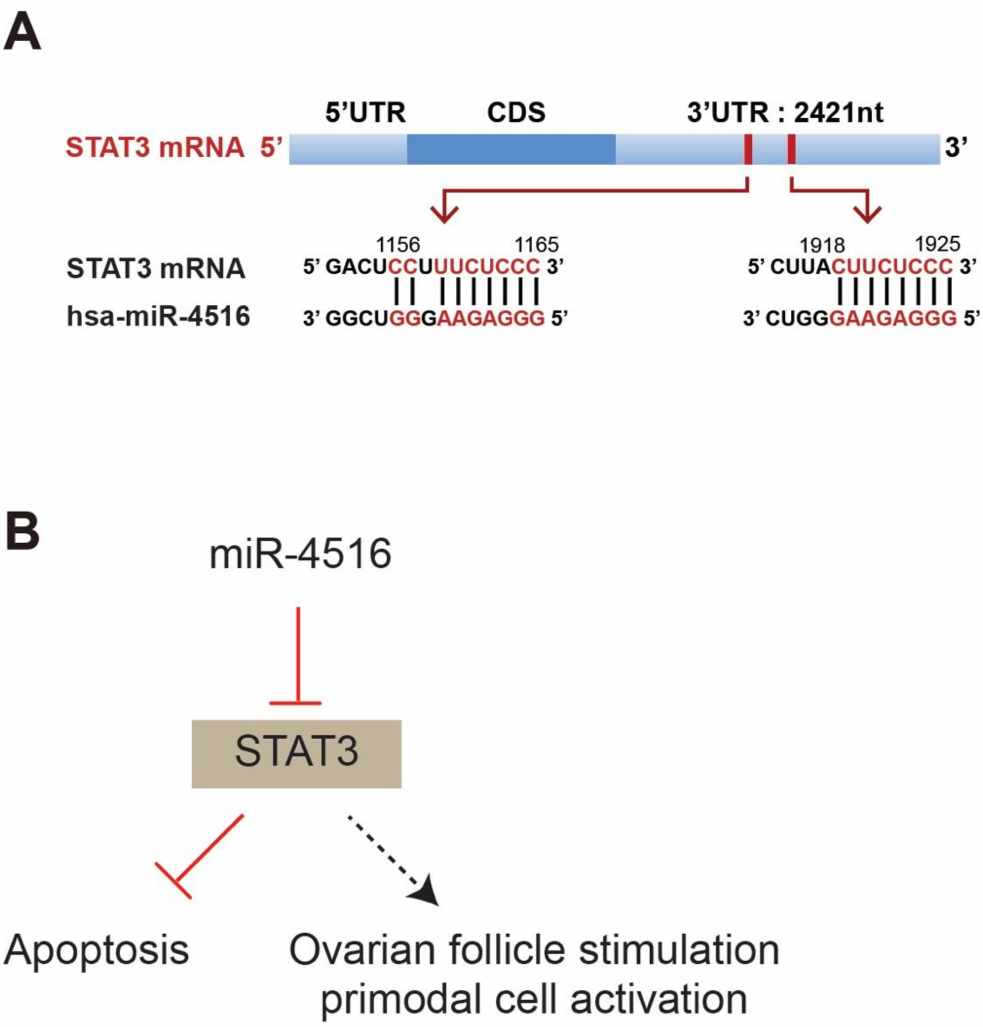

Figure S3. Predicted mechanism of the miR-4516-induced regulation of the STAT3 3′-UTR and ovarian function. (A) Schematic prediction of the binding sites of miR-4516 in the 3′-UTR of STAT3. (B) The anticipated role of miR-4516 in ovarian function via STAT3.

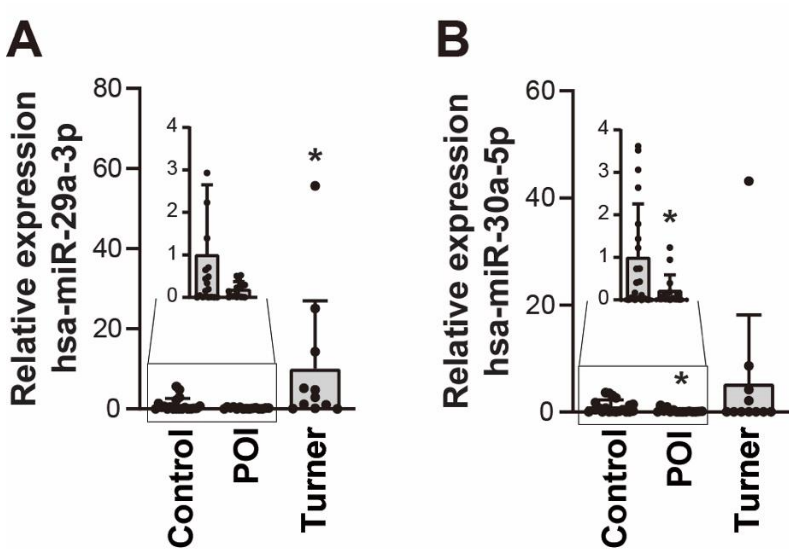

Figure S4. Relative expression of hsa-miR-29a-3p and hsa-miR-30a-5p in the validation cohorts.
Relative expression of hsa-miR-29a-3p (A) and hsa-miR-30a-5p (B) in the validation cohort using qRT-PCR (patients with premature ovarian insufficiency (POI) = 15, patients with Turner syndrome (Turner) = 11, control individuals = 20). Relative miRNA expression was normalized using UniSp6 as an internal control. Asterisks (*) indicate significant differences ($P<0.05$) when compared to the control group.
